# FragViz: visualization of fragmented networks

**DOI:** 10.1186/1471-2105-11-475

**Published:** 2010-09-22

**Authors:** Miha Štajdohar, Minca Mramor, Blaž Zupan, Janez Demšar

**Affiliations:** 1 Faculty of Computer and Information Science, University of Ljubljana, Slovenia; 2 Department of Molecular and Human Genetics, Baylor College of Medicine, Houston TX, USA

## Abstract

**Background:**

Researchers in systems biology use network visualization to summarize the results of their analysis. Such networks often include unconnected components, which popular network alignment algorithms place arbitrarily with respect to the rest of the network. This can lead to misinterpretations due to the proximity of otherwise unrelated elements.

**Results:**

We propose a new network layout optimization technique called FragViz which can incorporate additional information on relations between unconnected network components. It uses a two-step approach by first arranging the nodes within each of the components and then placing the components so that their proximity in the network corresponds to their relatedness. In the experimental study with the leukemia gene networks we demonstrate that FragViz can obtain network layouts which are more interpretable and hold additional information that could not be exposed using classical network layout optimization algorithms.

**Conclusions:**

Network visualization relies on computational techniques for proper placement of objects under consideration. These algorithms need to be fast so that they can be incorporated in responsive interfaces required by the explorative data analysis environments. Our layout optimization technique FragViz meets these requirements and specifically addresses the visualization of fragmented networks, for which standard algorithms do not consider similarities between unconnected components. The experiments confirmed the claims on speed and accuracy of the proposed solution.

## Background

From the onset of systems biology, visualization of networks has played a key role in communicating the relations between objects of interest and the structure of the problem domain. Gene networks [1], protein interactions [2,3], synergistic relations between SNPs [4], gene-based disease similarities [5], enzymatic relations and metabolic processes are just a few examples of domains where visualization of networks can aid in understanding the layout of the biological systems. The interest in this area has sparked the development of a large variety of software tools and approaches that deal with network layout optimization, data integration, interactive exploration of the networks and data analytics [6].

Formally, a network is a graph which consist of vertices (nodes) linked by edges. In systems biology, vertices can represent genes, proteins, metabolites, diseases, or other objects of interest. Edges abstract the relations between these objects.

The network often consists of a large number of unconnected components, like the recently published yeast protein interaction network [7] and a drug similarity network [8] with 160 and 240 unconnected components, respectively. Classical network layout techniques such as Fruchterman-Reingold [9], Kamada-Kawai [10] and Frick *et al. *[11] algorithms arrange unconnected components arbitrarily, which can wrongly suggest a relation between otherwise unrelated components.

**Figure 1:**
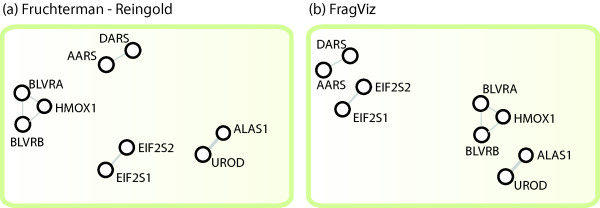
**Four components of the leukemia gene network from Figure 2**. The layout was optimized by a standard Fruchterman-Reingold algorithm (a) and by FragViz (b). FragViz optimization additionally used the information on vertex distances.

**Figure 2:**
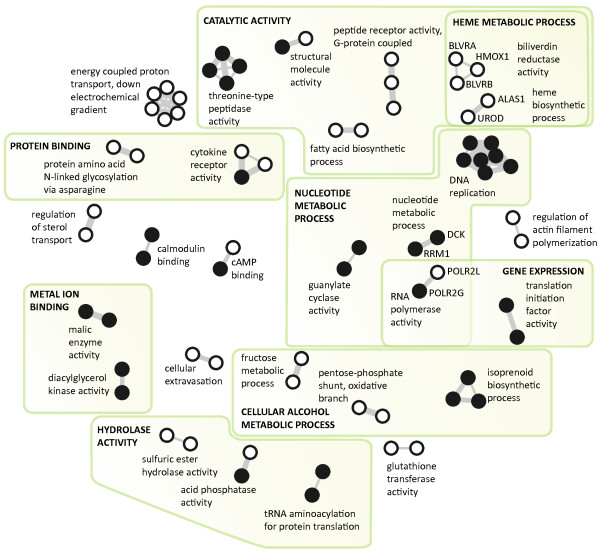
**The network (N1) of the significantly differentially expressed genes from the leukemia data set, where the similarity between the chosen genes was calculated based on their co-membership in biological pathways**. Only components with at least two vertices (similarity threshold equal to 0.7) are included in the network. Genes represented with solid circles were significantly over-expressed in the ALL samples and genes shown as empty circles had higher expression in the AML samples. The individual components are named according to the prevailing Gene Ontology annotation. Components were grouped and labeled manually by the expert.

For illustration consider the network from Figure 1, which depicts four components from the leukemia gene network shown in Figure 2. From the layout in Figure 1a with an arbitrary component placement one could (incorrectly) conclude that genes *blvra*-*hmox*-*blvrb *are more similar to *dars*-*aars *than to other genes in the graph. Misinterpretations like this can be avoided by displaying the network's main component, if one exists, separately, and then listing other (smaller) components. This type of display has been used, for instance, in the recently published disease gene network [5]. We discuss several other approaches and their shortcomings in related work.

In the paper we introduce a generally applicable technique called FragViz for placing the components according to the background data on their similarity. For example, rendering a network from Figure 1a. by our algorithm yields the layout in Figure 1b, from which we can infer that there is a relation between *blvra*-*hmox*-*blvrb *and *alas1*-*urod*. These are indeed correct relations as all of the mentioned genes have a function in heme metabolism. Notice that in the same visualization components *dars*-*aars *and *eif2s2*-*eif2s1 *are close to each other and all genes comprising them participate in protein translation. To render this network FragViz used additional information on mutual similarity between network nodes. It is clear that consideration of this additional information can improve the placement of unconnected components and expose additional information, thus avoiding misinterpretations based on the proximity of arbitrarily placed components.

FragViz uses a two-step network layout optimization procedure. It first applies the standard Fruchterman-Reingold algorithm separately on each unconnected component to optimize the layout of its vertices. Then it optimizes the global placement and orientation of components using a semi-physical model where the forces between components are inferred from similarities between the corresponding vertices in these components.

The data on similarity of the network nodes can either come from the same data source used to infer the structure of the network, or can be provided by supplying any additional information. Most often, the network's structure itself is derived from the *scored *relations between objects (*e.g*., the correlation in expression of two genes [12], the degree of SNP synergy in phenotype prediction [13], the number of disorder-specific genes shared by two diseases [14]). Edges then connect pairs of vertices for which the corresponding score exceeds some user-defined threshold. In such cases, the node pair similarity scores can be used as additional data for our procedure. If relations in the graph are not obtained by imposing thresholds on numerical data, other data source can be used to describe the vertex similarities. For instance, in the experimental study reported in the paper we show a protein-protein interaction network in which the vertex similarities are computed based on the biological function of the proteins.

### Related work

The proposed approach belongs to the family of algorithms for force-directed placement of objects into two-dimensional projections, and is strongly related to two kinds of algorithms: the optimization of network layout and multidimensional scaling (MDS) [15,16]. Network layout algorithms typically consider undirected graphs and optimize their layouts so that the pairs of connected vertices are placed closer to each other than to other vertices. If graph edges are weighed, shorter distances in the layout indicate stronger relations between objects represented with vertices. Multidimensional scaling considers an input matrix of object dissimilarities. It represents objects with points in Euclidean plane, and optimizes their placement so that the plotted distances match the dissimilarities as accurately as possible.

The two kinds of algorithms are related. It is possible to lay out a network by representing it with a distance matrix and performing MDS-based optimization. Or, vice versa, we can convert a distance matrix into a weighed complete graph and use a graph layout optimization in place of MDS. The optimizations would yield different results, as each of the methods uses its own stress function that is being optimized and was designed to match the goals of particular projection. For instance, in network layout optimization, projected distance between unconnected vertices has no effect for as long as it is large in comparison with distances between the connected vertices. In contrast, MDS optimizes distances between all pairs of objects, including the most distant.

With regard to the optimization procedure, algorithms make assumptions about the structure of the data. Network layout algorithms work best for graphs in which most vertices have only a small number of neighbors. MDS, on the contrary, considers distances between all pairs of objects, a data structure that can be represented with a complete weighed graph. Force-directed network layout optimization algorithms do not work well on densely connected graphs (*e.g*., [17]). The time complexity becomes prohibitive, and optimization may get trapped in the local optimum. In contrast, MDS is inapplicable to data with a large number of objects due to space complexity (prohibitively large distance matrix), whereas the Fruchterman-Reingold algorithm might still be useful if the number of edges is small enough.

There are a number of algorithms that use the metaphors from either network layout algorithms or MDS or both, trying to adapt each one for a particular data structure or heuristically improve runtime performance. Clustered graphs, for one, include groups of vertices that are related to each other. Clusters can be determined by observing the density of mutual connections between vertices or they can be based on data describing the vertices. Various algorithms have been designed that can detect such clusters [18-20]. Eades *et al. *[21] proposed a method for plotting clustered graphs, which models them in terms of four layers representing the entire graph, clusters, abridgments and pictures (groups of points shown in a particular projection). A corresponding model includes forces between connected vertices, between all vertices in each cluster, and between meta-vertices representing entire clusters. The performance of MDS can be improved by various heuristics. Morrison *et al. *[22], for instance, propose an algorithm which first projects a sample of points, then interpolates the remaining points between their positions, and finally fine-tunes the projection using a force model. These and similar methods can be used to speed up the layout optimization, increase the readability of the graph and construct user interfaces for interactive graph exploration. A complete survey of information visualization methods that focus on graph visualization techniques, can be found in [23].

The method described in this paper, FragViz, is a representative of context-specific methods for layout optimization. Unlike other methods we have reviewed in this section, it specifically addresses the layout optimization for graphs consisting of isolated components, which are given in advance and represent meaningful entities, such as groups of genes related to a particular process. The components, in turn, need to be considered jointly, based on their mutual relations which may stem from individual relations between member vertices. The natural approach that deals with this particular data structure is to first optimize the layout of each component independently, and then optimize the position and rotation of the components. We achieve this by combination of network layout and MDS-based algorithms. Notice that, as further addressed in the Discussion, other, perhaps more straightforward adaptations of existing approaches could address such data, but perform worse both in terms of runtime and quality of the resulting layout.

## Methods

The input to FragViz is a list of network components and a matrix of (dis)similarities between the network's vertices. FragViz first uses a network layout optimization technique, like Fruchterman-Reingold algorithm [9], to determine the placement of vertices within each of the connected components. Then, it finds a placement of components which reflect their mutual similarities. It is this second step that is an original contribution of our method, and which we in detail describe below.

Formally, we are given a graph *G *= (*V*, *E*) that consists of *p *disjunct components $V=\cup_{k=1}^{p}V_{k},$ and a |*V*| × |*V*| dissimilarity matrix *D*. The internal layout of each component *V*_*k *_is fixed and given by positions of its vertices inside its own fixed coordinate system. We will denote the position of vertex *v*_*i *_by **v**_**i**_. We also assume that the internal coordinate systems are centered, *i.e*. $\sum_{v_{i}\in V_{k}}v_{i}=0$ for each component *V*_*k*_. The task is to find the placement **c**_**k **_and orientation *ϕ*_*k *_of coordinate systems for all components, which reflect the given dissimilarities *D*.

### Description of a physical system

We will base the method on a physical metaphor. Imagine each component as a board with vertices as pegs. Pegs from different components are connected with springs of different lengths corresponding to the given dissimilarities *D*. The nature (or, in our case, a computer simulation) "optimizes" the system by finding the lowest energy configuration of the boards (components).

Assume that all vertices have equivalent mass *m*. The mass of the component *V*_*k *_is

(1) $$m_{k}=|V_{k}|m$$

and component's moment of inertia is

(2) $$I_{k}=m\sum_{v_{i}\in V_{k}}||v_{i}|{|}^{2}.$$

The force between a pair of points (*v*_*i*_, *v*_*j*_) is defined by Hooke's law,

(3) $$F_{ij}=(d_{ij}-||\;g_{i}-g_{j}\;||)\frac{g_{i}-g_{j}}{\left||\;g_{i}-g_{j}\;|\right|},$$

where **g**_**i **_and **g**_**j **_are positions of vertices in a global coordinate system,

(4) $$g_{i}=v_{i}+c_{k},$$

where *k *is such that *v*_*i *_∈ *V*_*k*_.

Let **F**_**i **_be the sum of forces acting on vertex *v*_*i*_

(5) $$F_{i}=\sum_{v_{j}\in V}F_{ij}.$$

The force causes linear acceleration

(6) $$a_{k}=\frac{\sum_{v_{i}\in V_{k}}F_{i}}{m_{k}}$$

and angular acceleration

(7) $$\alpha_{k}=\frac{\sum_{v_{i}\in V_{k}}F_{i}\times v_{i}}{I_{k}}$$

of the component. We shall assume infinite friction, so the component does not retain any momentum. At each instance, the component moves by a distance proportional to the linear acceleration, **Δc**_**k **_~ **a**_**k **_and rotates by an angle proportional to the angular acceleration, **Δ**ϕ_**k **_~ *α*_**k**_, so

(8) $$\Delta c_{k}\sim \frac{\sum_{v_{i}\in V_{k}}F_{i}}{\left|V_{k}\right|}$$

(9) $$\Delta \phi_{k}\sim \frac{\sum_{v_{i}\in V_{k}}F_{i}\times v_{i}}{\sum_{v_{i}\in V_{k}}||\;v_{i}\;|{|}^{2}}.$$

These equations allow for a computer simulation of the physical process. Starting from a random placement of components, we iteratively compute the forces **F**_**i**_, and move and rotate the components accordingly until the system reaches an optimum in which all **F**_**i **_are negligible.

### Approximate simulation

Computer simulation of the system described above is rather slow. We can speed it up by first computing the positions of components and then rotating them in place. The result is only approximately optimal with regard to the total stress (3), yet we will experimentally show that the difference is negligible.

For positioning the components, the approximate method measures and optimizes distances between components rather than the distances between vertices. We define the distance between components *V*_*k *_and *V*_*l *_as the average of distances between the corresponding vertices, similar to average linkage in hierarchical clustering analysis [24]:

(10) $$\delta_{kl}=\frac{1}{\left|\;V_{k}\;||\;V_{i}\;\right|}\sum_{\begin{array}{l} v_{i}\;\in \;V_{k}\\ v_{j}\in \;V_{l} \end{array}}d_{ij}.$$

The task is then to find the positions in a two dimensional plane, in which the distance between every pair of component centers **c**_**k **_and **c**_**l **_matches the given *δ*_*kl *_as close as possible. This approach is much faster than the simulation from the previous section since the computation of all pairwise distances at each step of optimization is replaced by a single such computation in (10). This translates the problem of placing the components into the familiar multidimensional scaling problem (MDS). There exist many efficient solutions of the MDS, such as, for instance, SMACOFF [25], which optimizes the overall energy of the system without computing its gradient, the force (3).

By considering only the centers of components, MDS ignores their sizes, which can cause the components to overlap. This can be fixed by introducing a scaling factor between the global coordinate system and the internal coordinate systems of components by replacing (4) by

(11) $$g_{i}=v_{i}+Kc_{k}.$$

The scaling factor is equal for all components and should be such that the components are just as large as possible without too much of overlap. A simple rule of a thumb is to use the ratio between the average size of components $\bar{v}$ and the average distance between them, $\bar{g},$ so

(12) $$K=\bar{v}/\bar{g}$$

where

(13) v¯=1p∑k=1p1|Vk |(|Vk |−1)∑vi,vj∈Vki≠j|| vi−vj ||

and

(14) $$\bar{g}=\frac{2}{p(p-1)}\sum_{k<l}||\;c_{k}-c_{l}\;||.$$

For rotation of components we use the original vertex-wise definition of force (3) computed in the scaled coordinate system (11). We apply the same procedure as in the exact simulation, except that we only compute the rotation without the translation. To avoid ending up in local minima, we use simulated annealing where the component can also rotate in the "wrong direction", with the probability of doing so decreasing with time. Although this optimization recomputes the pairwise distances between all vertices at each step, it is not overly time consuming since it requires only a small number of iterations.

In the remainder of the paper we only show layouts optimized by the approximate method.

### Data

The performance of the proposed algorithm was assessed on four different networks (N1, N2.1, N2.2 and N3) showing relations between genes which were most differentially expressed in the leukemia gene expression data set [26]. The original data set includes 4,860 genes whose expression was measured using DNA microarrays in 72 tissue samples classified either as acute lymphoblastic leukemia (ALL, 48 samples) or acute myeloid leukemia (AML, 25 samples). For N1, N2.1 and N2.2 we selected 1,025 differentially expressed genes with expression levels significantly smaller or larger (p-value < 0.01) according to Student's t-statistic with respect to the null distribution of the statistic. The null distribution was obtained by randomly permuting the class labels and calculating the *t*-statistic for all the genes. Network N3 was built with 131 out of 4,860 originally measured genes for which the information on their protein interactions was available in the MIPS mammalian protein-protein interaction database [27]. In the visualizations in the paper, genes represented with solid circles were significantly over-expressed in the ALL samples and genes shown as hollow circles had higher expression in the AML samples.

**Figure 3:**
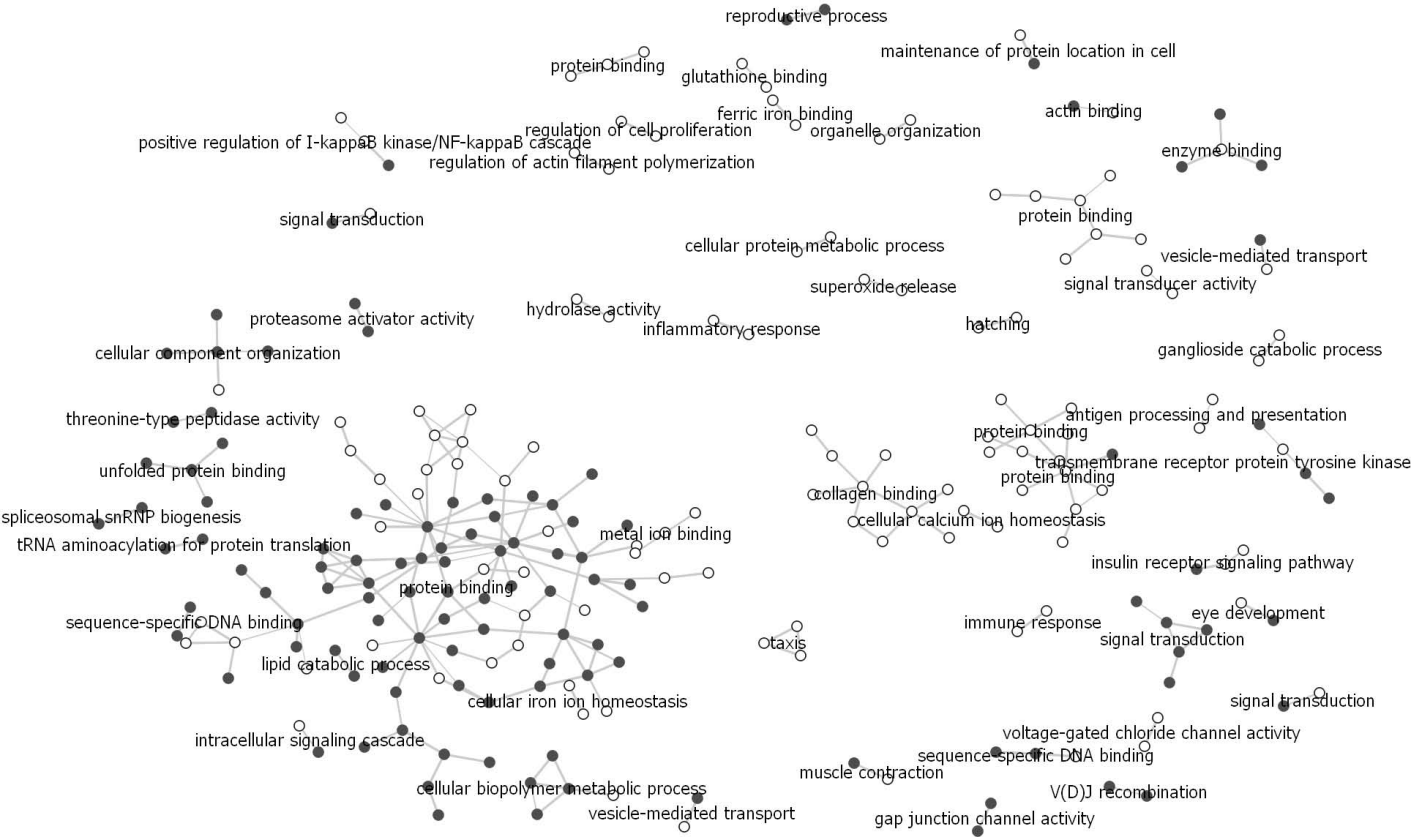
**The network (N2.1) of the most differentially expressed genes from the leukemia data set.** The similarity matrix of the chosen genes was taken from the recently published work of Huttenhower *et al*., 2009. The genes represented with solid circles were significantly over-expressed in acute lymphoblastic leukemia and the genes shown as empty circles had higher expression in acute myeloid leukemia.

**Figure 4:**
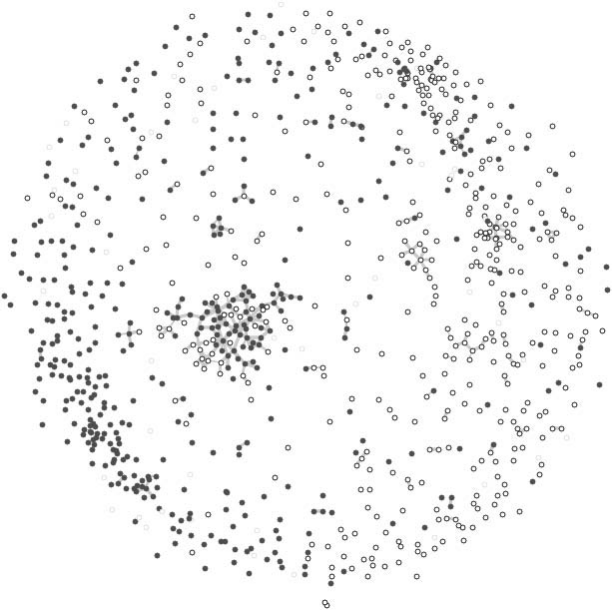
**The network (N2.2) of the most differentially expressed genes from the leukemia data set as the network in Figure 3, but including the isolated vertices (genes not connected to any other gene), in order to observe the similarity of all the differentially expressed genes**.

**Figure 5:**
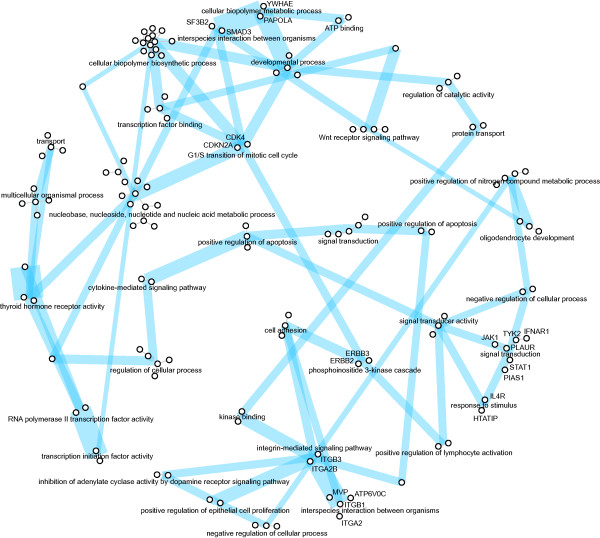
**The network (N3) of genes from the leukemia data set**. Vertices are connected based on their protein interactions from the MIPS database. Only the interactions with the confidence level equal to 1 are shown. The (dis)similarity matrix was added from a different data source and relates to genes biological functions. The individual components and their clusters are named according to the prevailing Gene Ontology annotation. Blue lines are drawn in the background, connecting each component with two most similar components. Line widths correspond to component similarity.

Based on different means to estimate the gene similarity, we have defined four distinct gene networks:

• N1 - biological function similarity score: the similarity of genes relates to their biological functions and was calculated based on their membership in canonical biological pathways using the Jaccard index [28]. The information on the membership of genes in biological pathways was acquired from the Molecular Signature Database [29] (C2 collection, canonical pathways). Figure 2 shows the network where the similarity threshold was set to 0.7 and all the unconnected genes were ignored.

• N2.1 - Huttenhower similarity score: the similarity between genes as computed by [30] using the information on all publicly available gene expression and protein interaction data, combined with prior knowledge from the Gene Ontology, KEGG, HPRD and other biological databases. Similarity scores above 0.999 for the leukemia genes were used to build the network. Only the genes connected to at least one other gene are included in the network (Figure 3).

• N2.2 - Huttenhower similarity score: the same similarity scores and threshold as in N2.1 were used (the Huttenhower *et al*., 2009 similarity score) for the N2.2 network. Differently to N2.1, N2.2 also includes isolated vertices (genes not connected to any other gene) in order to observe the similarity of all the differentially expressed genes (Figure 4).

• N3 - protein-protein interaction network (Figure 5): the leukemia genes were connected into the network based on their protein interactions from the MIPS mammalian protein-protein interaction database [27]. We additionally used the biological function similarity score (described under N1) for placing the interacting protein components based on the similar biological functions of the proteins comprising them.

The average local clustering coefficient [31] and the number of vertices, edges and components for these four networks are presented in Table 1. The local clustering coefficient of a vertex in a network quantifies how close its neighbors are to being a clique and describes the connectedness of a network.

**Table 1:** Basic characteristics of the networks used in experiments, describing the average local clustering coefficient and the number of vertices, edges and components

network	vertices	edges	components	**clustering coeff**.
N1	72	73	28	0.985
N2.1	240	223	54	0.864
N2.2	858	223	672	0.864
N3	132	121	41	0.852

## Results and Discussion

The goal of FragViz is to find the network layout in which the arrangement of components uncovers new insights on relations between them and their constituents. We evaluated the method in an experimental study that considered FragViz visualization of the leukemia gene networks N1, N2.1, N2.2 and N3. For additional assistance to the domain expert, the network components were named according to their most specific term from biological process or molecular function aspect of Gene Ontology [32].

### The leukemia gene network (N1)

Our goal was to obtain a clear visualization relating the most important genes and their biological functions for two major types of acute leukemia, yielding insight and valuable clues about the disrupted biological processes and pathways in leukemic cells. Solid vertices in Figure 2 represent genes significantly over-expressed in the ALL samples while empty circles are genes that had higher expression in the AML samples.

FragViz allows for the exploration of biological processes related to acute myeloid and acute lymphoblastic leukemia on different levels, from specific to more general ones. In Figure 2 additional Gene Ontology terms were assigned to groups of clusters which were determined manually by the expert to elucidate the disrupted biological pathways on a more general level as they cover higher number of differentially expressed genes. These ontological terms apply to all the genes in the marked areas and are significantly enriched with a p-value < 0.01. The components of the graph that are close to each other have similar biological and/or molecular functions according to Gene Ontology, demonstrating similarity between genes constituting them.

For example, the "guanylate cyclase activity", "nucleotide metabolic process", "RNA polymerase activity", and "DNA replication" components in Figure 2 all connect genes significantly over-expressed in acute lymphoblastic leukemia. All of these genes have a function in nucleotide metabolism and DNA biosynthesis. It is well known that lymphoblastic cells typically have severalfold higher activity of enzymes responsible for nucleotide metabolism enabling excessive proliferation of transformed cells [33]. Moreover, some of the pathways active in nucleotide metabolism, for example de novo purine synthesis (DNPS), have been recognized as important targets of antileukemic agents (*e. g*., methotrexate, mercaptopurine). In combination with other therapeutical agents, these drugs have improved survival of children with ALL to an overall cure rate of approximately 80 percent [34]. The network shown in Figure 2 clearly demonstrates this characteristic of acute lymphoblastic leukemia.

### The Huttenhower similarity network (N2.1 and N2.2)

The N1 and both N2 networks contain the same 1,025 differentially expressed genes from the leukemia data set. However, in N2.1 and N2.2 a combined gene distance score was used, computed from multiple biological data sources (*e.g*., gene expression, protein-protein interactions, biological function, ...) as proposed by Huttenhower *et al. *[30]. N2.1 shows only vertices with at least one edge. N2.2 also includes isolated vertices (genes not connected to any other gene), in order to observe the similarity of all the differentially expressed genes.

As in the N1 network, most of the graph components in N2 networks (Figures 3, 4) connect genes that are over-expressed in one of the two investigated kinds of leukemia (all genes in the component are the same color). One can observe that the genes significantly differentially expressed in the two investigated leukemias cluster together (Figures 3, 4). This reflects the well known phenomenon that not only individual genes, but whole processes and pathways are disrupted in cancer cells [35]. In Figure 4, the empty circles (AML) are clustered in the right part of the graph and the solid ones (ALL) in the left part, again demonstrating that expression changes in cancer tissues are disrupted on the level of pathways and processes.

For example, the genes in components "spliceosomal snRNP biogenesis", "tRNA aminoacylation for protein translation", "sequence-specific DNA binding" and the nearest genes in the component "protein binding" participate in processes of cell proliferation. All these genes have higher expression in ALL samples. Excessive cell proliferation is a characteristic of all leukemic cells. However, previous studies [36,37] have shown that the proliferative index of ALL cells is significantly higher compared to AML cells.

Since the distance information is used to adjust the position of unconnected components, the layout allows for the exploration of the data on different levels, using genes from a single component or from clusters of biologically related components.

### The protein-protein interaction network (N3)

The placement of unconnected components in a fragmented network can be optimized using the vertex distance information from a source other than that used in the inference of network structure. For example, the N3 network (Figure 5) shows the protein-protein interactions for the leukemia genes from the MIPS database. The network is fragmented into many smaller unconnected components. We used the biological function similarity score for calculating the similarities between the components and optimizing the network layout.

Several gene products (proteins) that lie close to each other in the FragViz optimized network (Figure 5) are actually in interaction, as is reported in Human Protein Reference Database (HPRD) [38], a different public repository that stores protein-protein interactions identified by experimental results. For example, in HPRD, the protein Integrin beta 3 (*itgb3 *) is in interaction with protein Integrin beta 1 (*itgb1 *). Also, proteins Poly A polymerase alpha (*papola*) and *smad3 *are both in interaction with protein *smad2*. According to HPRD, protein interactions also exist among proteins in the components *il4r*-*htatip *and the near-lying component in the optimized layout. To outline them in the network, the vertices that correspond to these proteins (in Figure 5) are labeled accordingly. While our goals was not to use network layout optimization for protein interaction prediction, the cases mentioned here demonstrate the potential utility of different data sources in network layout optimization.

We added an optional component similarity visualization to the network. The similarity between network components is visualized by blue lines in Figure 5. Each component is connected to two most similar components and the line width represents the magnitude of the similarity. In Figure 5, most connected components are placed close to each other. However, in few cases similar components are positioned apart. Besides the technical problem - the optimization getting stuck in a local optimum - this may happen when two components belonging to different clusters of components nevertheless share a common function or when some component essentially belongs to two clusters. For example, genes in the component "G1/S transition of mitotic cell cycle" influence gene expression, as do most of the genes in the nearest cluster of components. The same component however also participates in the apoptotic pathway which is reflected in its connection with the "phosphoinositide 3-kinase cascade" component, a representative of components related to the apoptotic processes.

### Performance comparison

Table 2 compares the running times of simulation for six different layout optimization approaches: Fruchterman-Reingold algorithm, the exact and approximated method of FragViz, MDS and two applications of clustered graph approach, proposed by Eades *et al. *[21]. Clustered graph visualization method is applied on a graph *G′ *and a cluster tree *T′*. We transformed the original graph *G *and a dissimilarity matrix *D *to a clustered graph *C′ *= (*G′*, *T′*) in two different ways. In the first approach (denoted Eades 1), the component structure (*G*) was embedded in a two-level cluster tree (root-components-data objects) *T′ *in which every component from *G *represents a cluster in the first level of *T′*. Object dissimilarities *D *were used as weights in a complete graph *G′*. In the second approach (Eades 2), the original graph was retained (*G′ *= *G*) and a hierarchical clustering method was applied on dissimilarity matrix *D *to construct a cluster tree *T′*. Four different linkage functions were tested (average, single, complete and Ward's linkage). Since they all produced similar results, we report only on the performance of average linkage. For N3, the MDS was run on the dissimilarity matrix data. We used the standard SMACOFF algorithm for MDS; an exhaustive comparison of various heuristic enhancements is beyond the topic of this paper.

**Table 2:** Average layout optimization time in seconds for all four networks

network	FR	FragViz (simulation)	FragViz (approximation)	MDS	Eades 1	Eades 2
N1	0.4	33	6	36	3	1
N2.1	1.3	63	6	64	31	2
N2.2	8	301	240	320	410	29
N3	1.1	76	14	55	8	1.5

All measurements have been conducted on a desktop PC, with Intel Core 2 Duo 2.20GHz processor and 4 GB of RAM, using the 64-bit Windows 7 OS. The results represent an average over 10,000 runs of the algorithms on the N1-N3 networks, starting from random positions of vertices.

The Fruchterman-Reingold algorithm is by far the fastest, but it uses less data than the others and the resulting projections are much less informative. Running times of Eades 2 are comparable to those of Fruchterman-Reingold. This was expected, as both approaches run on a similar graph. Eades 1 employs a complete graph, which makes it much slower. On large networks, Eades 1 (N2.2) is even slower than MDS. The running times of FragViz simulation are similar to those of MDS, which is also expected. The approximate method runs much faster, except for the large network N2.2, where most vertices are unconnected, which essentially translates the visualization problem to MDS.

Table 3 compares the quality of layouts in terms of Pearson correlation between the vertex similarities and their distances in the projection. Although the approximate method runs much faster than the simulation, the decrease in quality is small. Moreover, the approximate method sometimes outperforms the exact one, which suggests that the optimization can get trapped in a local minimum.

**Table 3:** Pearson's correlation between elements of the gene distance matrix and the Euclidean distance between the corresponding vertices in the two-dimensional network layout

network	FR	Fragviz (simulation)	FragViz (approximation)	MDS	random	Eades 1	Eades 2
N1	0.311	0.391	0.380	0.415	0.007	0.173	0.215
N2.1	0.086	0.290	0.302	0.654	0.002	0.009	0.156
N2.2	0.401	0.591	0.609	0.593	0.006	0.391	0.043
N3	0.179	0.224	0.285	0.361	0.060	0.092	0.199

For all four networks, the correlation coefficients of the FragViz algorithms are very similar. The correlation was always lower with the FR algorithm and, for three out of four networks, the highest correlation was obtained with MDS. In one of the compared networks (N2.2) MDS performed slightly worse than approximation, suggesting MDS got trapped in a local minimum. As expected, when the vertices were arbitrary placed in the graph, the correlation between the position of vertices in the graph and their actual distances is close to 0.

Clustered graph approaches (Eades 1 and Eades 2) are in general faster than FragViz, but performed worse in terms of layout quality. Eades 2 performed better than Eades 1 on smaller graphs (N1, N2.1 and N3), whereas Eades 1 had a high correlation for a large network (N2.2). However, Eades 1 approach is not appropriate for analyzing large fragmented networks as it works prohibitively slow.

Note that the compared algorithms pursue different goals. The tests were run on data suitable for the method presented in this paper, while in other contexts another method could give better results. In particular, clustered graph methods could not be directly applied to the original data, so its results depend on the proposed transformation of the original problem.

### Impact of the network fragmentation

**Figure 6:**
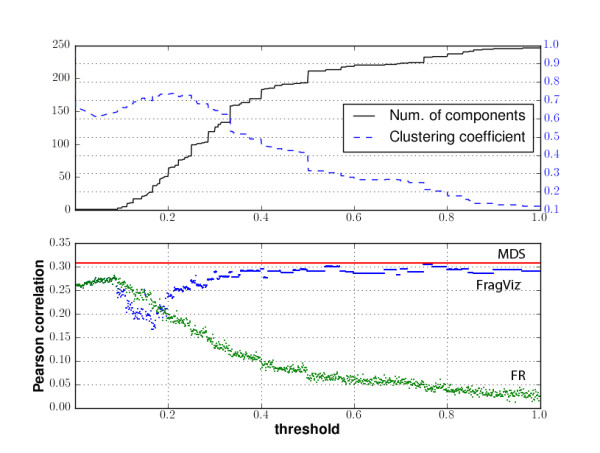
**Influence of the selected similarity threshold on the layout optimization**. Biological function similarity score was used on input. Horizontal axis measures the connectedness of the network, where 0 represents a complete graph and 1 means the graph has no edges.

We also investigated the behavior of layout optimization methods with respect to the degree of network fragmentation. We constructed 1,000 networks of the most differentially expressed genes from the leukemia data set (visualized in Figure 2) with similarity threshold required for an edge from 0.0 (the graph is fully connected) to 1.0 (no edges between vertices). Figure 6a shows the correlations between the network layout and the (dis)similarities matrix for the FR, MDS and FragViz algorithms. Figure 6b shows how the average local clustering coefficient [31] and the number of components change with different threshold values.

FragViz and FR algorithms are equivalent when the network consists of only one component (threshold values lower than 0.1). For the FR algorithm, the correlation decreases when the network gets more fragmented. However, when the fragmentation increases (threshold value greater than 0.2), the correlation score of the FragViz algorithm increases and rises above the best score obtained by the FR algorithm. Correlation for MDS does not depend on the threshold.

### Alternative approaches

**Figure 7:**
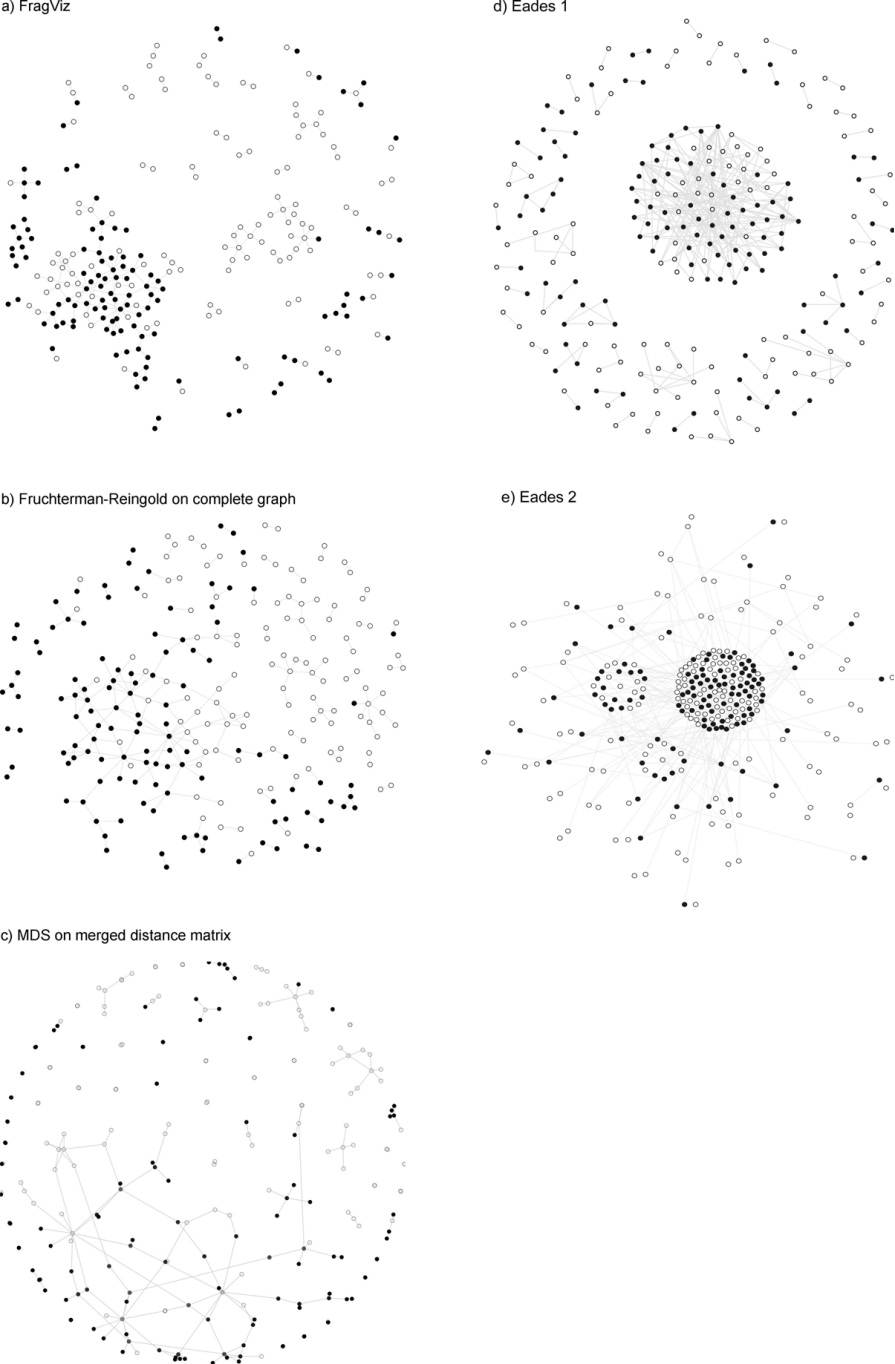
**The N2.1 network layout optimized with four different methods, two different approaches were used for clustered graph visualization**. In 7.a the network was optimized with the FragViz algorithm. For 7.b a complete weighted graph was first constructed from the original network and similarity matrix. The weights of the network edges were scaled so that the largest weight equalled 1. Virtual edges were added to all unconnected pairs of vertices, with weights inversely linear with the distances from the similarity matrix and scaled to interval [0, 0.01]. The complete graph was then optimized with the FR algorithm. For 7.c the original network was merged to the dissimilarity matrix, where pairs of connected vertices from the original network had the lowest value in the similarity network 0, while other values from the dissimilarity matrix were 100 times smaller [0.99, 1]. The dissimilarity matrix was than optimized with the MDS algorithm. In 7.d and 7.e we optimized a network using clustered graph visualization. We transformed the original graph *G *and dissimilarity matrix *D *to a clustered graph *C′ *= (*G′*, *T′*) in two different ways.

Projections similar to those by FragViz could in principle be obtained with other algorithms (Figure 7). The graph can be augmented with virtual (hidden) edges with small weights corresponding to the distances between the vertices and then optimized using graph layout optimization algorithms. Alternatively, we can construct a distance matrix in which the distances combine a term representing the graph edge (*e.g*. 0 for connected objects and 1 for unconnected) and the term from the original distance matrix, scaled to have only a minor influence. Such combinations are, though, inefficient. First, force-based optimization techniques often get stuck in local optima. Graphically, if optimizing the entire picture at once, they are unable to pull together the vertices belonging to the same component if too many other, unrelated vertices are randomly scattered between them and push them apart. We discovered that using the two standard algorithms, Fruchterman-Reingold algorithm and the SMACOFF algorithm for MDS, in such manner consistently fail to optimize the projection in quite common cases where the network includes components with more than 15 vertices. A typical example is shown in Figure 7c. Our two-step procedure avoids that problem by first composing the components.

Besides the projection quality issues, FragViz is also faster than the above approaches since it splits the optimization problem into a set of much smaller problems, laying out small individual components and then arranging a small number of components instead of all vertices at once. Using the graph layout optimization algorithms instead of FragViz, as described above, would be slower since these algorithms do not perform well on complete graphs. For MDS, to get similar running times as FragViz, one needs to employ fast heuristic MDS algorithms, which gain speed by somewhat compromising the quality of the projection [22].

Figures 7d and 7e show some shortcomings of cluster based approaches on this particular data. When the problem is transformed so that the cluster structure is defined by graph components and applied over the complete graph (Eades 1), the optimization is more likely to end up in a local minimum due to a higher number of forces involved. In Figure 7d, we cannot spot any regions containing mostly solid or empty vertices, as opposed to Figures 7a and 7b by FragViz and by Fruchterman-Reingold algorithms. This may also be one of the reasons behind the worse Pearson correlations of this approach in general (Table 2). The second way in which we used cluster based layout optimization, Eades 2, gives better correlations and running times, yet the resulting layouts are visually unsatisfactory: the cluster structure does not correspond exactly to the graph components, so the vertices belonging to the same component may be pulled apart since they ended up in different clusters. We were unable to alleviate this problem by tweaking the parameters of the method.

## Conclusions

We have recently witnessed the emergence of large repositories of biomedical research and clinical data. Methods are needed that would allow the domain experts to sieve through these data sets to gather information, reason on the hidden patterns and form plausible hypotheses to be tested in subsequent studies. Here, visualization combined with visual data analytics plays a major role, as it can reveal the data patterns and allow the experts to explore the data.

Visualizations require the development of dedicated algorithms that craft the proper placement of the object under consideration. Explorative data analysis requests these to be fast to be able to construct responsive interfaces. We have developed a layout optimization technique FragViz that meets these requirements and specifically addresses the visualization of fragmented networks, where standard algorithms do not consider similarities between unconnected components.

FragViz is neither faster than all existing algorithms nor more accurate in terms of the match between the given and the projected distances. FragViz is slower than the Fructherman-Reingold algorithm, which is a direct consequence of considering more information. The resulting vertex distances may match the given distance matrix worse than in multidimensional scaling, a consequence of fixing the layout of the components. This is a matter of design decision: the goal of FragViz is to provide a sensible local picture and a global overview, thence the two level optimization. It can happen, for instance, that in a chain-like component the two vertices on the edge are weakly related to a common third vertex not belonging to the component. While other layout optimization algorithms would bend the chain, FragViz keeps it straight. Our experiments confirmed usefulness of the proposed solution. The case study on the leukemia gene networks shows that derived visualizations may be helpful in uncovering the relations between the components.

The data, networks, their visualizations, and the implementation of the described methods in an open-source analytics framework Orange [39] are available on the supplementary web page at http://www.ailab.si/supp/fragviz. An online network optimization web application is available at http://www.ailab.si/fragviz.

## Availability and Requirements

**Project name: **Orange FragViz

**Project home page: **http://www.ailab.si/orange

**Operating system: **Platform independent

**Programming language: **Python, C++

**Other requirements: **PyQt, PyQwt, Numpy

**License: **GNU GPL

**Any restrictions on use by non-academics: **none

## Authors' contributions

BZ identified the problem and suggested its solution. MS developed and implemented the algorithm, performed the experiments and drafted the manuscript. MM designed and interpreted the case study. JD formulated the optimization problem based on the physical metaphor. All authors co-wrote the article and approved its final version.
